# Locus coeruleus contrast and diffusivity metrics differentially relate to age and memory performance

**DOI:** 10.1038/s41598-024-66238-z

**Published:** 2024-07-04

**Authors:** Ilana J. Bennett, Jason Langley, Andrew Sun, Kitzia Solis, Aaron R. Seitz, Xiaoping P. Hu

**Affiliations:** 1 Department of Psychology, University of California, 900 University Avenue, 2127 Psychology Building, Riverside, CA 92521-0426 USA; 2 Center for Advanced Neuroimaging, University of California Riverside, Riverside, CA USA; 3 Department of Psychology, Northeastern University, Boston, MA USA; 4 Department of Bioengineering, University of California Riverside, Riverside, CA USA

**Keywords:** Long-term memory, Cognitive ageing, Cognitive neuroscience, Neural ageing

## Abstract

Neurocognitive aging researchers are increasingly focused on the locus coeruleus, a neuromodulatory brainstem structure that degrades with age. With this rapid growth, the field will benefit from consensus regarding which magnetic resonance imaging (MRI) metrics of locus coeruleus structure are most sensitive to age and cognition. To address this need, the current study acquired magnetization transfer- and diffusion-weighted MRI images in younger and older adults who also completed a free recall memory task. Results revealed significantly larger differences between younger and older adults for maximum than average magnetization transfer-weighted contrast (MTC), axial than mean or radial single-tensor diffusivity (DTI), and free than restricted multi-compartment diffusion (NODDI) metrics in the locus coeruleus; with maximum MTC being the best predictor of age group. Age effects for all imaging modalities interacted with sex, with larger age group differences in males than females for MTC and NODDI metrics. Age group differences also varied across locus coeruleus subdivision for DTI and NODDI metrics, and across locus coeruleus hemispheres for MTC. Within older adults, however, there were no significant effects of age on MTC or DTI metrics, only an interaction between age and sex for free diffusion. Finally, independent of age and sex, higher restricted diffusion in the locus coeruleus was significantly related to better (lower) recall variability, but not mean recall. Whereas MTC has been widely used in the literature, our comparison between the average and maximum MTC metrics, inclusion of DTI and NODDI metrics, and breakdowns by locus coeruleus subdivision and hemisphere make important and novel contributions to our understanding of the aging of locus coeruleus structure.

## Introduction

The locus coeruleus has garnered significant attention in neurocognitive aging research in recent years given that it is one of the first brain regions to accumulate tau pathology implicated in Alzheimer’s Disease^1,2^. Even in the absence of disease, histopathological studies in humans have found reductions in locus coeruleus neurons with age^3–6^ c.f.^7^. These noradrenergic neurons project throughout the brain^8–10^ and are thought to play a neuromodulatory role in a broad range of cognitive processes^11–15^, including memory. Whereas recent advances in magnetic resonance imaging (MRI) have made it possible to reliably image and segment this small brainstem structure in vivo^16–18^, this rapidly growing field will benefit from consensus regarding which metrics of locus coeruleus structure are most sensitive to age and cognitive performance.

Structural “integrity” of the locus coeruleus is most often assessed using either fast spin-echo T1-weighted or magnetization transfer-weighted MRI sequences in which signal intensity (or contrast) is thought to be driven by the magnetic properties of neuromelanin^17,19–22^ c.f.^23^, which is a pigmented byproduct of norepinephrine synthesis in the locus coeruleus. Magnetization transfer contrast (MTC) ratio in the locus coeruleus is measured relative to the pons and then either averaged across voxels within an anatomical mask of the locus coeruleus (e.g.,^24^) or extracted from the voxel(s) with the maximum value (e.g.,^2,25,26^). However, concerns have been raised about whether maximum MTC accurately captures the locus coeruleus^27^ as age-related neuronal loss may be unequally distributed across the structure, comparable to what is seen in Alzheimer’s Disease^28^.

Using these MRI approaches, cross-sectional studies have consistently found a quadratic or inverted U-shaped function in locus coeruleus MTC across the adult lifespan. Locus coeruleus MTC increases with age from 20 to ~ 60 years and decreases with age after ~ 60 years old^2,24,29,30^, although some studies within only older adults have found no effect of age on locus coeruleus MTC^31,32^. Results have also been mixed when comparing extreme age groups, with some studies reporting higher locus coeruleus MTC in older than younger adults^25,33^, and others finding no age group differences^34–37^. There is some evidence that these age effects are more prominent in the rostral than caudal subdivision of the locus coeruleus^24,35,38,39^, consistent with the rostral subdivision being more vulnerable to cell loss in aging and Alzheimer’s Disease^28^. Of the handful of studies that have examined sex difference in locus coeruleus MTC, most have reported no significant sex effects^2,24,29,31^, whereas at least one observed lower locus coeruleus MTC in females than males that was independent of age group^25^. There is little evidence that these age group differences in MTC vary in the left and right hemisphere^39,40^.

In contrast to MTC, there has been a dearth of literature using other approaches that can assess microstructural “integrity” of the locus coeruleus, such as diffusion-weighted MRI. When diffusion of molecular water in each voxel is modelled using a single-tensor, diffusion tensor imaging (DTI) metrics such as mean diffusivity (MD) measure the average rate of diffusion, which varies with tissue properties including neurodegeneration, neuroinflammation, and an accumulation of pathology. A small group of recent studies have revealed lower diffusivity in older than younger adults in locus coeruleus gray matter^37,41,42^ c.f.^33^. To date, however, no studies have assessed effects of age on the locus coeruleus using multi-compartment diffusion approaches, such as Neurite Orientation Dispersion and Density Imaging (NODDI)^43^. NODDI may be better suited than DTI for capturing microstructural properties in gray matter as it models compartments of diffusion that are invariant to tissue organization, yielding stronger effects of age and cognition in regions such as the hippocampus^44^. Moreover, although some of these studies assessed locus coeruleus using both DTI and MTC^33,37^, they did not compare the sensitivity of these imaging modalities to age.

Age-related degradation of the locus coeruleus, as measured by either MTC or diffusion (DTI, NODDI), would have significant consequences for cognitive processes mediated by brain regions innervated by its noradrenergic projections, such as memory. Consistent with this view, previous studies have shown that lower MTC^2,12,35,36,45,46^ and lower DTI diffusivity^41,42^ in locus coeruleus gray matter relates to worse memory performance in older adults. For diffusivity, these relationships have been observed in both rostral and caudal locus coeruleus^42^. Whereas these studies have focused on average memory performance (e.g., mean across trials), a measure of variability between trials may be more sensitive to locus coeruleus structure as any degradation may result in momentary disruptions to attention via locus coeruleus neuromodulation, resulting in less consistent performance^47^.

The current study sought to address the gaps of prior work by acquiring magnetization transfer- and diffusion-weighted MRI images in younger and older adults who also completed a word list free recall memory task. Effects of age on locus coeruleus structure were assessed by comparing extreme age groups (younger, older) as well as relationships to age within older adults for MTC (average, maximum), DTI (mean diffusivity, MD; axial diffusivity, AD; radial diffusivity, RD), and NODDI (restricted, free) metrics. Effects of sex (male, female), subdivision (rostral, caudal), and hemisphere (left, right) on aging of locus coeruleus structure were also examined, as were relationships between locus coeruleus structure and memory performance using measures of both mean recall and recall variability.

## Methods

### Participants

Fifty-eight younger and 87 older adults who were recruited from the University of California, Riverside and surrounding communities met our inclusion criteria, which included having normal general cognition (e.g., < 17 on the telephone Montreal Cognitive Assessment, MoCA;^48^), self-reported absence of major health conditions (e.g., stroke, dementia, diabetes); and being free of conditions that would prevent them from being able to enter the MRI scanner (e.g., non-MR compliant implants, difficulty lying in the supine position, claustrophobia). One younger adult was excluded after data collection due to uncorrectable mis-registration that yielded inaccurate MRI metrics.

The final sample consisted of 57 younger (18–26 years) and 82 older (60–87 years) adults. Demographic and neuropsychological data are provided in Table 1, excluding all or partial demographic (5 younger, 3 older), telephone MoCA (maximum score 22; 5 older), and free recall (3 younger, 12 older) data because responses were not recorded or tasks were not completed.

**Table 1:** Demographic and neuropsychological test data.

	Younger	Older	*t*/*χ*^2^	*r*
N	57	82	n/a	n/a
Age (years)	20.1 ± 2.1	69.0 ± 5.9	**57.9**	n/a
Sex (% female)	55.5%	64.6%	1.1	n/a
Education (years)	13.6 ± 1.5	15.6 ± 2.7	**4.8**	< 0.01
Ethnicity (% Non-Hispanic)	71.2%	84.4%	3.3	n/a
Race (% White)	28.8%	77.6%	**30.1**	n/a
Handedness (% right-handed)	94.2%	100.0%	5.5	n/a
MoCA	n/a	19.9 ± 1.5	n/a	− **0.41**
Free recall mean	5.1 ± 1.3	4.8 ± 1.4	− 1.1	− 0.22
Free recall variability	0.2 ± 0.1	0.3 ± 0.2	1.3	0.08

Mean and standard deviation (M ± SD) or percent (%) scores are provided for each age group. Significant age group differences at *p* < 0.05 are indicated by bolded *t* (mean scores) or *χ*^2^ (% scores) statistics. Within the older age group, significant correlations with chronological age at *p* < 0.05 are indicated by bolded *r* statistics.

All participants provided informed consent and received course credit or financial compensation for participation. Study procedures were approved by the Institutional Review Board of the University of California, Riverside and all experimental procedures were performed in accordance with the approved guidelines and regulations.

### Magnetic resonance imaging data

#### Acquisition

Imaging data were acquired on a 3 T MRI scanner (Prisma, Siemens Healthineers, Malvern, PA) at the Center for Advanced Neuroimaging at the University of California, Riverside. Excitation was performed using the body coil on the scanner and signal was received using a 32-channel receive only coil.

A T_1_-weighted MP-RAGE image was acquired with the following parameters: echo time (TE)/repetition time (TR)/inversion time = 3.02/2600/800 ms, GRAPPA acceleration factor = 2, flip angle = 8°, voxel size = 0.8 × 0.8 × 0.8 mm^3^.

Magnetization transfer-prepared gradient echo (MT-GRE) images were acquired with the following parameters: TE/TR = 3.21/385 ms, flip angle = 40°, field of view (FOV) = 220 × 186 mm^2^, matrix size = 512 × 432, slice thickness = 3 mm, magnetization transfer preparation pulse (flip angle = 370°, 1.5 kHz off-resonance, duration = 10 ms), 4 averages. Slices were positioned perpendicular to the dorsal edge of the brainstem at the midline along the fourth ventricle.

Diffusion-weighted single-shot spin-echo, echo planar images were acquired with the following parameters: TE/TR = 75/4100 ms, FOV = 202 × 170 mm^2^, matrix size = 176 × 148, voxel size = 1.15 × 1.15 × 2.5 mm^3^, and 32 slices with no gap. Slices were aligned parallel to the hippocampus and covered the brain from the middle of the cerebellum to the striatum. Monopolar diffusion-encoding gradients were applied in 30 directions with *b* values of 500 s/mm^2^ and 2000s/mm^2^. Two sets of *b* = 0 images were acquired, with the two sets having opposite polarities of phase-encoding direction for the correction of susceptibility distortion^49^.

#### Regions of interest

**Figure 1:**
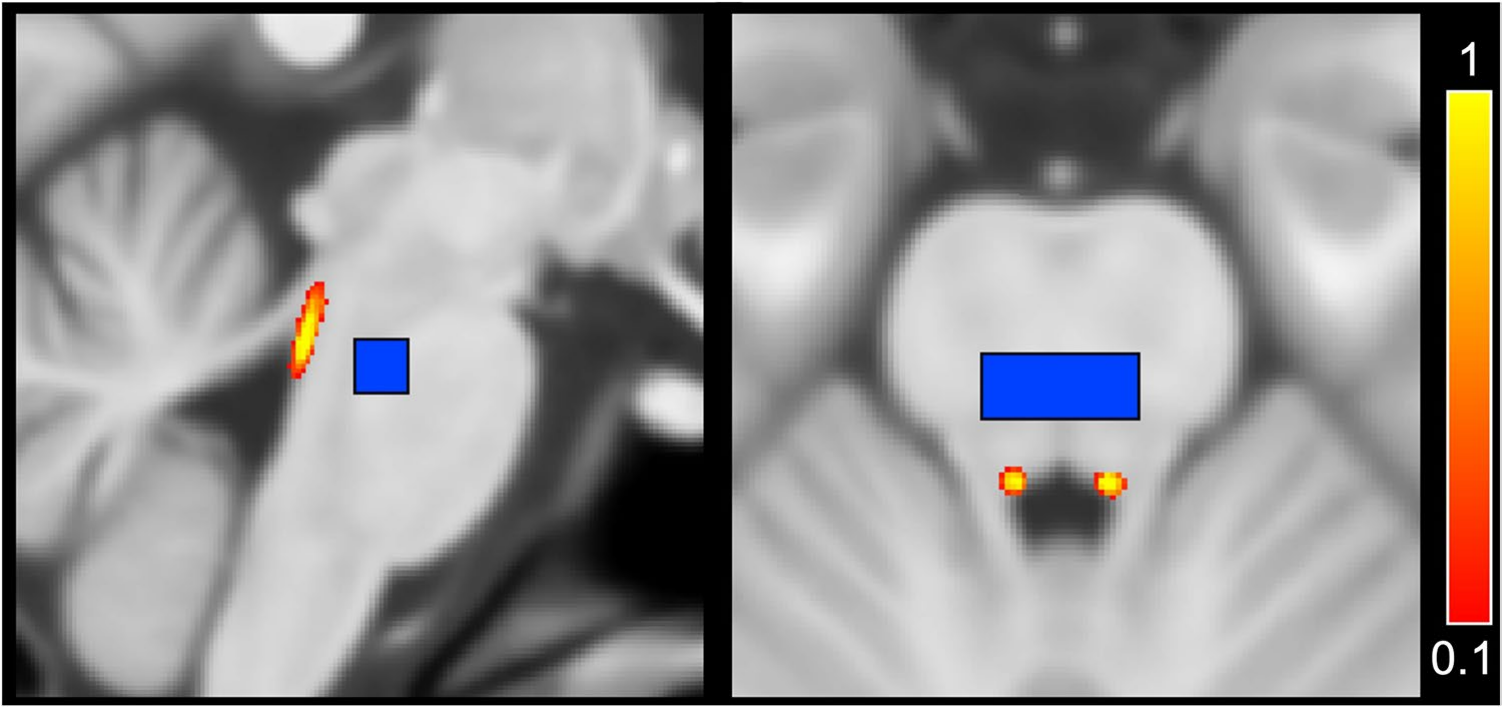
Sagittal (left) and axial (right) views of the atlas-based bilateral locus coeruleus (red-yellow) and rectangular midline pontine reference region (blue) in Montreal Neurological Institute (MNI) T_1_-weighted space.

A standardized atlas was used to define bilateral locus coeruleus, as well as its rostral and caudal subdivisions^41^. A rectangular midline reference region was defined in the pons (Fig. 1). For each participant, regions of interest were aligned from Montreal Neurological Institute (MNI) space to their MT-GRE and diffusion-weighted space using a transformation that concatenated an alignment between MNI T_1_-weighted space and their T_1_-weighted image using FMRIB’s Linear Image Registration Tool (FLIRT) and FMRIB’s Nonlinear Image Registration Tool (FNIRT) in the FMRIB Software Library (FSL)^50^ and an alignment between the participant’s T_1_-weighted and either their MT-GRE (using the averaged MT-GRE image) or diffusion-weighted (using the average* b* = 0 image) space using separate rigid body transformations with a boundary-based registration cost function. Each aligned region of interest was thresholded at 50% and binarized.

#### MTC metrics

For each participant, individual measurements from the MT-GRE acquisition were corrected for motion by registering them to the first image using a FLIRT rigid-body transformation and then averaged. Finally, a magnetization transfer contrast (MTC) image was calculated using the following equation:

$$MTC=\left({I-I}_{ref}\right)/{I}_{ref}$$

where *I* denotes the intensity of a voxel in the MT-GRE image and *I*_*ref*_ is the mean intensity of the pontine reference region. Average and maximum MTC metrics were calculated by averaging MTC values or finding the peak MTC value within the MT-GRE-aligned locus coeruleus regions of interest, respectively. Individual values were excluded from the MTC analyses if they exceeded four standard deviations from the sample mean (2 older).

#### Diffusion metrics

For each participant, raw diffusion-weighted data were corrected for motion, susceptibility distortions, and eddy currents using FSL’s TOPUP and EDDY^49,51^. Diffusion tensor imaging (DTI) metrics (mean diffusivity, MD; axial diffusivity, AD; radial diffusivity, RD) were estimate using the *b* = 0 and 500 data with FSL’s DTIFIT. Neurite orientation dispersion and density imaging (NODDI) metrics (free diffusion, also known as ‘fiso’; restricted diffusion, also known as ‘ficvf’) were estimated using the *b* = 0, 500, and 2000 data with NODDI toolbox v1.0.1 in MATLAB^43^. Mean diffusion metrics were calculated by averaging values within the diffusion-aligned locus coeruleus regions of interest. Eight participants (3 younger, 5 older) were excluded from all NODDI analyses for having free diffusion values of zero, indicating poor model fit in the first stage. Individual values were also excluded from the DTI (1 younger) and NODDI (2 younger) analyses if they exceeded four standard deviations from the sample mean.

#### Volume metrics

Locus coeruleus volume estimates and its relation to age, sex, and memory performance are reported in the Supplementary Information (Supplementary Fig. 1).

### Memory test

Participants completed three unique trials of a free recall task. On each trial, participants repeat out loud each of 10 words that were shown one at a time on a computer screen (30 s total), followed by an immediate free recall test (90 s). Words were selected from the Auditory Verbal Learning Test^52^ and did not repeat across trials. Mean recall was calculated as the average number of words recalled across trials. Recall variability (coefficient of variation) was calculated as the standard deviation across trials divided by the mean^53^. Twelve participants (3 younger, 12 older) were excluded from these analyses because they did not complete the memory task.

### Statistical approach

Age group differences on all demographic and memory performance metrics were first assessed in all participants using either between-group *t*-tests for mean scores or *χ*^2^ tests for percentile scores. Effects of age on all demographic and memory performance metrics were then assessed within older adults using correlations with chronological age.

Effects of age and sex on locus coeruleus structure were first assessed in all participants separately for each imaging modality (MTC, DTI, NODDI) using Age Group (younger, older) × Sex (male, female) × Metric (MTC: average, maximum; DTI: MD, AD, RD; NODDI: restricted, free) mixed factorial ANOVAs with values from the whole locus coeruleus. To assess whether these effects differed across locus coeruleus subdivisions or hemispheres, separate Age Group × Sex × Metric × Subdivision (rostral, caudal) and Age Group × Sex × Metric × Hemisphere (left, right) mixed factorial ANOVAs were conducted for each imaging modality. Maximum MTC was excluded from the latter ANOVAs as it appeared in just one subdivision or hemisphere for each participant. Significant interactions were probed using independent sample *t*-tests for each level of the independent variable(s) and these post-hoc comparisons were not corrected for multiple comparisons. Stepwise logistic regressions were then used to identify the locus coeruleus metric(s) that best predicted age group. One model included all metrics from the whole locus coeruleus and sex as predictor variables entered with Forward Wald selection. Additional model used all metrics from either the locus coeruleus subdivisions or hemispheres and sex as predictor variables.

Effects of age and sex on locus coeruleus structure were next assessed within older adults using multiple regression analyses that were conducted separately using values from the whole locus coeruleus for each metric from each imaging modality. For each model, chronological Age, Sex, and their interaction were predictor variables and Metric was the observed variable. Additional regression analyses were then conducted using values from each locus coeruleus subdivision or hemisphere. These analyses were not corrected for multiple comparisons as they were not repeated on the same dependent measure. Comparable analyses were not conducted in younger adults due to their restricted age range.

The extent to which locus coeruleus structure related to memory performance was assessed in all participants using separate partial correlations between each memory metric (mean recall, recall variability) and each metric from each imaging modality in the whole locus coeruleus, controlling for age and sex. Additional correlation analyses were then conducted using values from each locus coeruleus subdivision and hemisphere. Significant effects survived Bonferroni correction for two comparisons per imaging metric (*p* < 0.025).

The extent to which locus coeruleus structure metrics were related was assessed in all participants using separate partial correlations among each metric from each imaging modality in the whole locus coeruleus, controlling for age and sex. Significant effects survived Bonferroni correction for six comparisons per imaging metric (*p* < 0.008).

## Results

### Demographic differences between younger and older adults

Tests for age group differences on demographic and memory performance metrics in all participants revealed that more older adults self-identified as White, *χ*^2^(2, *N* = 128) = 30.1, *p* < 0.001, and completed more years of education, *t*(132) = 57.9, *p* < 0.001, than younger adults. These data are presented in Table 1. Effects of controlling for race and education are provided in the Supplementary Information (Supplementary Tables 1 and 2).

Within older adults, correlations revealed that older age was significantly related to worse MoCA performance, *r* = − 0.41, *p* < 0.001.

### Locus coeruleus structure differs between younger and older adults

**Figure 2:**
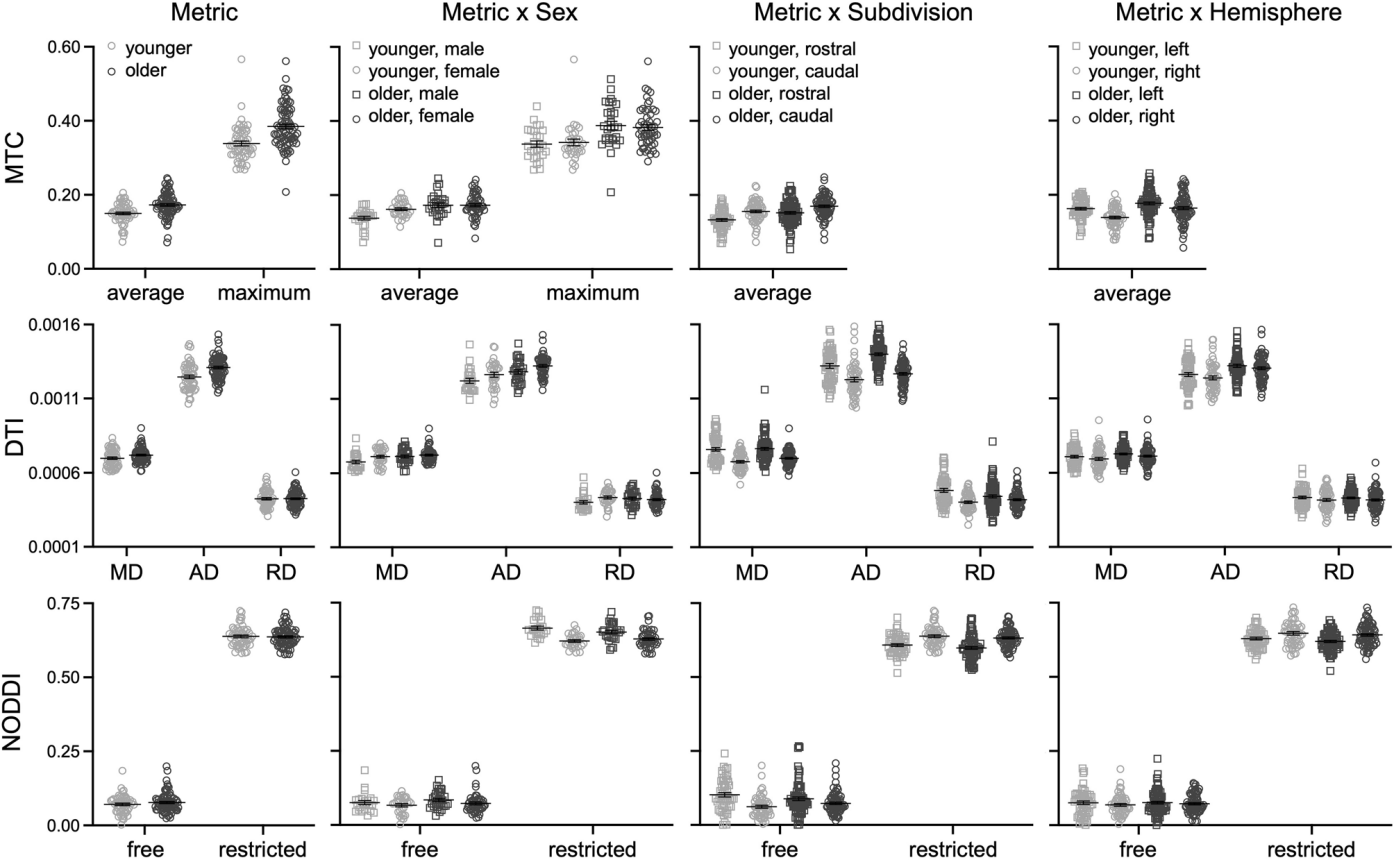
Age group differences in locus coeruleus structure are shown for each imaging metric (first column), with additional breakdowns by sex, subdivision, and hemisphere.

Effects of age group and sex on locus coeruleus structure in all participants are presented in Tables 2, 3, 4 and 5 and Fig. 2.

**Table 2:** Locus coeruleus ANOVA results.

	MTC			DTI			NODDI		
	Whole	Subdiv^a^	Hemis^a^	Whole	Subdiv^a^	Hemis^a^	Whole	Subdiv^a^	Hemis^a^
Age group	**33.2**	**15.2**	**16.8**	**10.2**	**5.5**	**10.1**	0.5	0.2	0.2
Metric	**1968.7**	n/a	n/a	**13269.8**	**16163.7**	**12236.2**	**29737.2**	**21923.5**	**30665.1**
Age group × metric	**6.9**	n/a	n/a	**14.5**	**28.4**	**13.9**	**4.4**	**4.2**	**5.9**
Sex	1.0	2.6	3.5	**6.7**	**7.6**	**6.7**	**21.1**	**12.1**	**22.0**
Sex × age group	1.6	**6.2**	**5.3**	1.2	1.2	1.1	0.4	< 0.1	0.6
Sex × metric	2.4	n/a	n/a	**3.4**	**4.7**	**3.8**	**14.6**	**9.8**	**14.4**
Sex × age group × metric	1.0	n/a	n/a	2.1	**3.0**	2.3	4.1	**5.5**	**4.4**
Subdivision	n/a	**68.5**	n/a	n/a	**119.5**	n/a	n/a	0.8	n/a
Subdivision × age group	n/a	1.1	n/a	n/a	0.3	n/a	n/a	3.8	n/a
Subdivision × metric	n/a	n/a	n/a	n/a	**42.9**	n/a	n/a	**123.6**	n/a
Subdivision × age group × metric	n/a	n/a	n/a	n/a	**23.6**	n/a	n/a	3.2	n/a
Subdivision × sex	n/a	< 0.1	n/a	n/a	< 0.1	n/a	n/a	1.4	n/a
Subdivision × sex × age group	n/a	0.3	n/a	n/a	1.0	n/a	n/a	**6.4**	n/a
Subdivision × sex × metric	n/a	n/a	n/a	n/a	0.2	n/a	n/a	0.1	n/a
Subdivision × sex × age group × metric	n/a	n/a	n/a	n/a	0.1	n/a	n/a	1.4	n/a
Hemisphere	n/a	n/a	**72.0**	n/a	n/a	**11.8**	n/a	n/a	**11.7**
Hemisphere × age group	n/a	n/a	**6.9**	n/a	n/a	0.1	n/a	n/a	< 0.1
Hemisphere × metric	n/a	n/a	n/a	n/a	n/a	0.2	n/a	n/a	**43.5**
Hemisphere × age group × metric	n/a	n/a	n/a	n/a	n/a	0.4	n/a	n/a	0.1
Hemisphere × sex	n/a	n/a	0.4	n/a	n/a	0.5	n/a	n/a	2.1
Hemisphere × sex × age group	n/a	n/a	0.1	n/a	n/a	0.1	n/a	n/a	1.0
Hemisphere × sex × metric	n/a	n/a	n/a	n/a	n/a	0.8	n/a	n/a	3.1
Hemisphere × sex × age group × metric	n/a	n/a	n/a	n/a	n/a	0.4	n/a	n/a	< 0.1

*F* statistics are provided for each ANOVA run using values from either the whole locus coeruleus (whole), its subdivisions (subdiv), or its hemispheres (hemis) for each dependent metric (MTC, DTI, NODDI). Significant effects at *p* < 0.05 are bolded.

^a^There are no effects of Metric for the subdivision and hemisphere analyses that were only run using average MTC because the maximum value appeared in just one subdivision or hemisphere.

**Table 3:** Whole locus coeruleus ANOVA descriptive statistics.

	Younger		Older	
	Male	Female	Male	Female
MTC metrics				
Average	0.14 ± 0.006	0.16 ± 0.005	0.17 ± 0.005	0.17 ± 0.004
Maximum	0.34 ± 0.011	0.34 ± 0.010	0.39 ± 0.010	0.38 ± 0.008
DTI metrics^u^				
MD	0.68 ± 0.010	0.71 ± 0.009	0.71 ± 0.009	0.72 ± 0.007
AD	1.22 ± 0.016	1.26 ± 0.015	1.28 ± 0.015	1.32 ± 0.011
RD	0.40 ± 0.011	0.44 ± 0.010	0.43 ± 0.010	0.42 ± 0.007
NODDI metrics				
Restricted	0.66 ± 0.006	0.62 ± 0.005	0.65 ± 0.005	0.63 ± 0.004
Free	0.07 ± 0.007	0.07 ± 0.006	0.09 ± 0.006	0.07 ± 0.004

Mean ± standard error are provided for each dependent metric (MTC, DTI, NODDI) in the whole locus coeruleus, separately for each age and sex group.

^u^units × 10^−3^.

**Table 4:** Locus coeruleus subdivision ANOVA descriptive statistics.

	Younger				Older			
	Male		Female		Male		Female	
	Rostral	Caudal	Rostral	Caudal	Rostral	Caudal	Rostral	Caudal
MTC metrics								
Average	0.12 ± 0.006	0.15 ± 0.006	0.14 ± 0.005	0.16 ± 0.005	0.16 ± 0.006	0.17 ± 0.005	0.15 ± 0.004	0.17 ± 0.004
DTI metrics^u^								
MD	0.73 ± 0.016	0.66 ± 0.011	0.77 ± 0.015	0.69 ± 0.010	0.76 ± 0.015	0.69 ± 0.010	0.76 ± 0.011	0.71 ± 0.007
AD	1.28 ± 0.019	1.20 ± 0.018	1.33 ± 0.017	1.24 ± 0.016	1.34 ± 0.018	1.23 ± 0.017	1.41 ± 0.013	1.23 ± 0.012
RD	0.45 ± 0.018	0.39 ± 0.011	0.50 ± 0.016	0.42 ± 0.010	0.45 ± 0.017	0.42 ± 0.011	0.44 ± 0.012	0.42 ± 0.008
NODDI metrics								
Restricted	0.63 ± 0.008	0.66 ± 0.006	0.60 ± 0.007	0.62 ± 0.005	0.61 ± 0.007	0.64 ± 0.005	0.59 ± 0.005	0.63 ± 0.004
Free	0.09 ± 0.011	0.06 ± 0.007	0.11 ± 0.010	0.05 ± 0.006	0.10 ± 0.010	0.08 ± 0.006	0.08 ± 0.007	0.07 ± 0.005

Mean ± standard error are provided for each dependent metric (MTC, DTI, NODDI) in each locus coeruleus subdivision, separately for each age and sex group.

^u^units × 10^−3^.

**Table 5:** Locus coeruleus hemisphere ANOVA descriptive statistics.

	Younger				Older			
	Male		Female		Male		Female	
	Left	Right	Left	Right	Left	Right	Left	Right
MTC metrics								
Average	0.15 ± 0.006	0.13 ± 0.007	0.17 ± 0.005	0.15 ± 0.006	0.18 ± 0.006	0.17 ± 0.006	0.18 ± 0.004	0.16 ± 0.004
DTI metrics^u^								
MD	0.69 ± 0.011	0.67 ± 0.012	0.72 ± 0.010	0.71 ± 0.011	0.73 ± 0.010	0.70 ± 0.011	0.73 ± 0.008	0.72 ± 0.008
AD	1.24 ± 0.020	1.21 ± 0.018	1.27 ± 0.018	1.25 ± 0.016	1.29 ± 0.018	1.28 ± 0.016	1.33 ± 0.013	1.32 ± 0.012
RD	0.41 ± 0.012	0.40 ± 0.013	0.44 ± 0.011	0.43 ± 0.012	0.44 ± 0.011	0.42 ± 0.012	0.42 ± 0.008	0.42 ± 0.008
NODDI metrics								
Restricted	0.65 ± 0.006	0.68 ± 0.007	0.62 ± 0.006	0.63 ± 0.006	0.63 ± 0.005	0.66 ± 0.006	0.61 ± 0.004	0.63 ± 0.005
Free	0.07 ± 0.008	0.07 ± 0.007	0.07 ± 0.008	0.06 ± 0.006	0.08 ± 0.007	0.08 ± 0.006	0.07 ± 0.006	0.07 ± 0.004

Mean ± standard error are provided for each dependent metric (MTC, DTI, NODDI) in each locus coeruleus hemisphere (left, right), separately for each age and sex group.

^u^units × 10^−3^.

#### MTC metrics

##### MTC age group differences

The whole locus coeruleus analysis revealed significant effects of Age Group and Metric, with higher MTC in older than younger adults and for the maximum than average metric. A significant Age Group × Metric interaction revealed that the age group difference was larger for the maximum (mean difference = M_diff_: 0.048) than average (M_diff_: 0.024) metric.

##### MTC age group differences varied by sex

The whole locus coeruleus ANOVA revealed no significant effects with Sex. The subdivision and hemisphere ANOVAs conducted only on the average MTC metric further revealed significant Sex × Age Group interactions, with higher average MTC in older than younger males (M_diff_: 0.029), but not females (M_diff_: 0.006).

##### MTC age group differences did not vary by subdivision

The locus coeruleus subdivision ANOVA revealed a significant main effect of Subdivision, with higher average MTC in the caudal than rostral subdivision. However, there were no significant interactions between Subdivision and Age Group.

##### MTC age group differences varied by hemisphere

The locus coeruleus hemisphere ANOVA revealed significant effects of Hemisphere and Hemisphere × Age Group, with higher average MTC in the left than right hemisphere, but with the age group difference (higher average MTC in older than younger adults) being significantly larger in the right (M_diff_: 0.027) than left (M_diff_: 0.015) hemisphere.

#### DTI metrics

##### DTI age group differences

The whole locus coeruleus analysis revealed significant effects of Age Group and Metric, with higher diffusivity in older than younger adults for AD, then MD, and then RD. A significant Age Group × Metric interaction revealed that the age group difference was larger for AD (M_diff_: 0.062 × 10^−3^) than MD (M_diff_: 0.024 × 10^−3^), but not significant for RD (M_diff_: 0.006 × 10^−3^).

##### DTI age group differences varied by sex

The whole locus coeruleus analysis revealed significant effects of Sex and Sex × Metric with higher diffusivity in females than males and this sex difference was significant for AD (M_diff_: 0.040 × 10^−3^) and MD (M_diff_: 0.022 × 10^−3^), but not RD (M_diff_: 0.013 × 10^−3^). However, there were no significant interactions between Sex and Age Group.

The locus coeruleus subdivision ANOVA did reveal a significant Sex × Age Group × Metric interaction, which was probed using separate independent sample *t*-tests for each metric in each sex group. Results revealed significantly higher AD in older than younger males (M_diff_: 0.062; *p* = 0.003) and females (M_diff_: 0.066; *p* < 0.001); significantly higher RD in younger than older females (M_diff_: − 0.030; *p* = 0.036), but not males (M_diff_: 0.013; *p* = 0.443); and a non-significant trend for higher MD in older than younger males (M_diff_: 0.029; *p* = 0.059), but not females (M_diff_: 0.017; *p* = 0.890).

##### DTI age group differences varied by subdivision

The locus coeruleus subdivision ANOVA revealed significant effects of Subdivision, Subdivision × Metric, and Subdivision × Age Group × Metric. The three-way interaction was probed using separate independent sample *t*-tests for each metric in each subdivision, which revealed higher diffusivity in older than younger adults for rostral AD (M_diff_: 0.086 × 10^−3^; *p* < 0.001), caudal AD (M_diff_: 0.041 × 10^−3^; *p* = 0.011), and caudal MD (M_diff_: 0.025 × 10^−3^; *p* = 0.009); higher diffusivity in younger than older adults for rostral RD (M_diff_: − 0.033 × 10^−3^; *p* = 0.038); and no significant age group difference was significant for rostral MD (M_diff_: 0.007 × 10^−3^; *p* = 0.650) or caudal RD (M_diff_: 0.017 × 10^−3^; *p* = 0.100).

##### DTI age group differences did not vary by hemisphere

The locus coeruleus hemisphere ANOVA revealed a significant main effect of Hemisphere, with higher diffusivity in the left than right hemisphere. However, there were no significant interactions between Hemisphere and Age Group.

#### NODDI metrics

##### NODDI age group differences

The whole locus coeruleus ANOVA revealed a significant Age Group × Metric interaction, with a non-significant trend for higher diffusion in older than younger adults for the free (M_diff_: 0.010; *p* = 0.080), but not restricted (M_diff_: − 0.004; *p* = 0.467), metric. A significant main effect of Metric revealed higher restricted than free diffusion.

##### NODDI age group differences varied by sex

The whole locus coeruleus ANOVA revealed significant Sex and Sex × Metric effects, with higher diffusion in males than females and this sex difference was significant for restricted (M_diff_: 0.033), but not free (M_diff_: 0.008), diffusion.

The locus coeruleus subdivision and hemisphere ANOVAs did reveal significant Sex × Age Group × Metric interactions, which were probed using separate independent sample *t*-tests for each metric in each sex group. Results revealed significantly higher restricted diffusion in younger than older males (M_diff_: 0.017; *p* = 0.037), but not females (M_diff_: 0.002; *p* = 0.826), and not for free diffusion in either males (M_diff_: − 0.015; *p* = 0.112) or females (M_diff_: 0.002; *p* = 0.661).

##### NODDI age group differences varied by subdivision

The locus coeruleus subdivision ANOVA revealed significant effects of Subdivision × Metric and Subdivision × Sex × Age Group. The three-way interaction was probed using separate independent sample *t*-tests for each sex group in each subdivision, which revealed non-significant trends for higher diffusion in older than younger females in the caudal subdivision (M_diff_: − 0.011; *p* = 0.066) and in younger than older females in the rostral subdivision (M_diff_: 0.016; *p* = 0.057), but not in males in either the caudal M_diff_: 0.003; *p* = 0.668) or rostral (M_diff_: < 0.001; *p* = 0.965) subdivision.

##### NODDI age group differences did not vary by hemisphere

The locus coeruleus hemisphere ANOVA revealed significant effect of Hemisphere and Hemisphere × Metric, with higher diffusion in the right than left hemisphere for restricted (M_diff_: 0.023), but not free (M_diff_: − 0.004), metric. However, there were no significant interactions between Hemisphere and Age Group.

#### Best predictor of age group

The whole locus coeruleus stepwise logistic regression revealed that maximum MTC was the best predictor of age group, *x*^2^(1) = 28.2, *p* < 0.001, correctly classifying 73.4% of participants. Classification accuracy was slightly improved when adding AD (78.2%) or AD and average MTC (81.5%) to the model.

The locus coeruleus subdivision stepwise logistic regression revealed that rostral AD was the best predictor of age group, *x*^2^(1) = 22.4, *p* < 0.001, correctly classifying 69.9% of participants. Classification accuracy was slightly improved when adding rostral RD (76.4%); rostral and caudal RD (78.9%); or rostral and caudal RD and caudal AD (79.7%) to the model.

The locus coeruleus hemisphere stepwise logistic regression revealed that right hemisphere AD was the best predictor of age group, *x*^2^(1) = 21.0, *p* < 0.001, correctly classifying 70.4% of participants. Classification accuracy was slightly improved when adding right hemisphere average MTC (75.2%) to the model.

### Locus coeruleus structure does not differ within older adults

**Figure 3:**
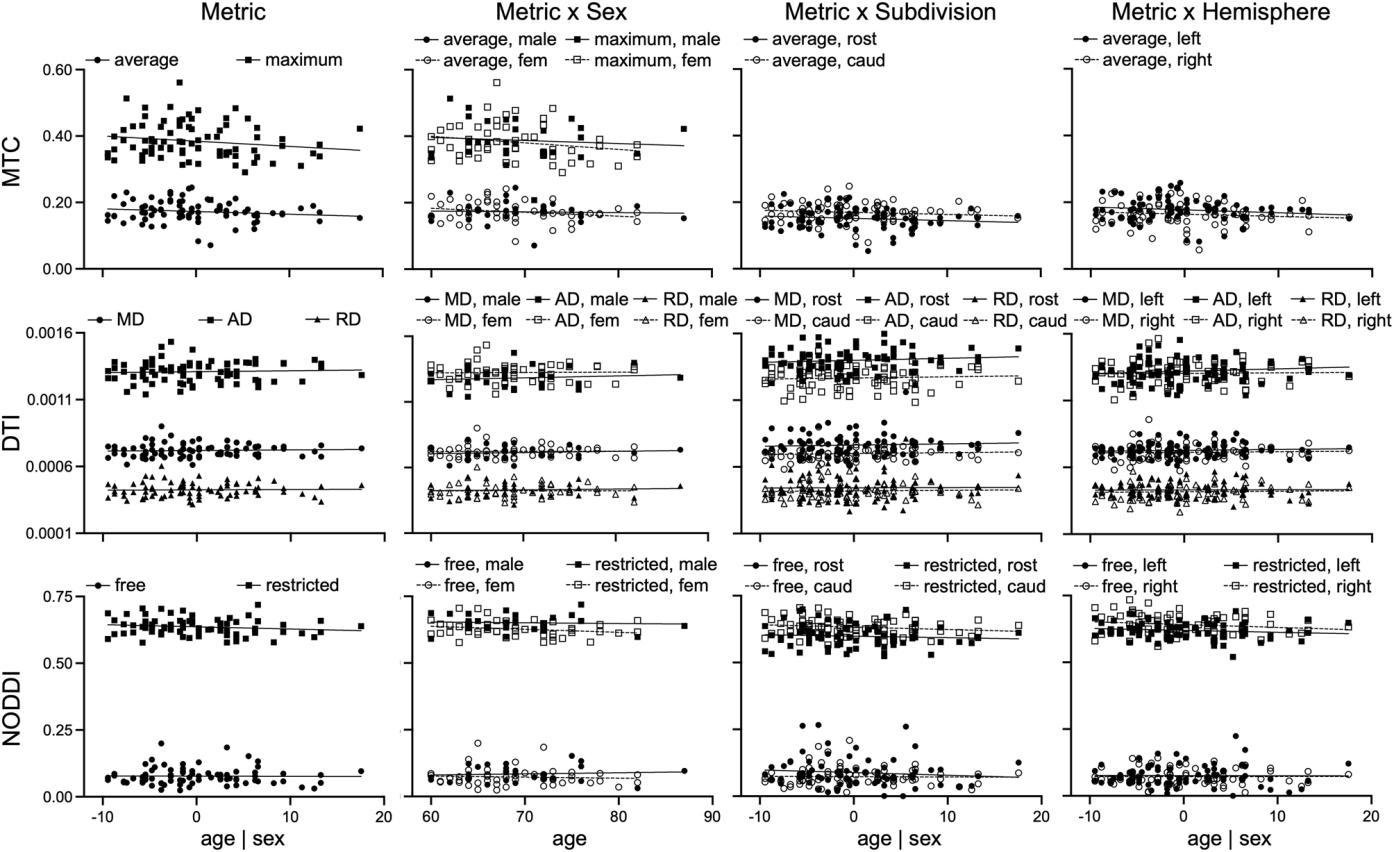
Relationships between chronological age and locus coeruleus structure within older adults are shown for each imaging metric (first column), with additional breakdowns by sex, subdivision, and hemisphere.

Effects of age and sex on locus coeruleus structure within older adults are presented in Table 6 and Fig. 3. The whole locus coeruleus and locus coeruleus subdivision regression analyses revealed no significant effects. The locus coeruleus hemisphere regression analyses revealed significant effects of Sex and Age Group × Sex for free diffusion in the left hemisphere, with older age relating to higher free diffusion in males and lower free diffusion in females.

**Table 6:** Relationships to age within older adults.

	MTC			DTI			NODDI	
	Average	Maximum^a^	MD		AD	RD	Restricted	Free
Whole locus coeruleus								
Age	− 0.22	− 0.20	0.12		0.02	< 0.01	− 0.21	− 0.07
Sex	− 0.99	− 0.56	− 0.64		− 0.78	− 0.31	− 0.54	− 0.68
Age × sex	1.00	0.62	0.55		0.52	0.38	0.92	0.86
Rostral subdivision								
Age	− 0.22	n/a	− 0.04		0.10	− 0.10	− 0.11	− 0.17
Sex	− 1.35	n/a	− 1.40		− 0.19	− 1.44	− 0.19	− 0.69
Age × sex	1.45	n/a	1.46		> − 0.01	1.57	0.45	0.89
Caudal subdivision								
Age	− 0.10	n/a	0.09		0.05	0.06	− 0.20	− 0.06
Sex	0.48	n/a	− 0.08		− 0.63	0.25	− 0.11	− 0.58
Age × sex	− 0.42	n/a	− 0.13		0.27	− 0.23	0.40	0.69
Left hemisphere								
Age	− 0.16	n/a	− 0.02		0.07	− 0.08	− 0.21	− 0.21
Sex	− 0.11	n/a	− 1.38		− 0.68	− 1.32	− 0.80	− **2.68**
Age × sex	0.12	n/a	1.37		0.45	1.51	1.05	**2.85**
Right hemisphere								
Age	− 0.22	n/a	0.07		0.02	0.07	− 0.16	0.06
Sex	− 1.63	n/a	0.22		− 0.32	0.49	0.45	0.91
Age × sex	1.71	n/a	− 0.35		0.07	− 0.51	− 0.12	− 0.78

Standardized *Beta* coefficients are provided for each regression run on either the whole locus coeruleus, its subdivisions (rostral, caudal), and hemispheres (left, right) for each dependent metric (MTC, DTI, NODDI). Significant effects at *p* < 0.05 are bolded.

^a^Maximum MTC only has whole locus coeruleus results because the maximum value appeared in just one subdivision or hemisphere.

### Locus coeruleus structure relates to memory performance

**Figure 4:**
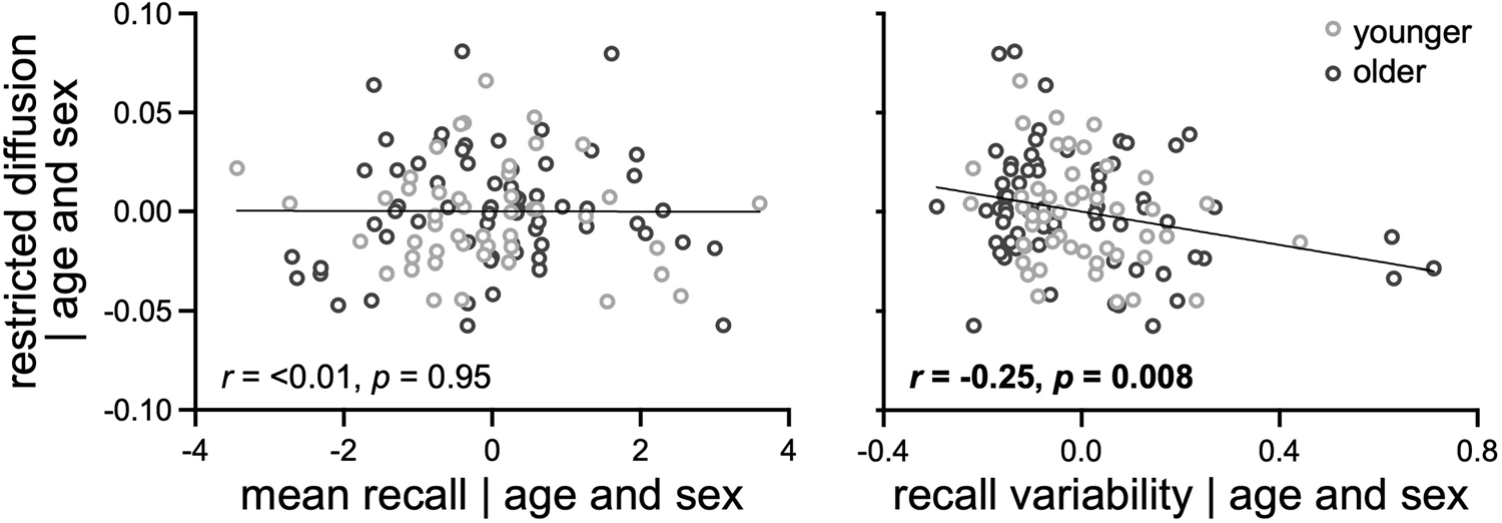
Better (lower) recall variability, but not mean recall, significantly related to higher restricted diffusion in whole locus coeruleus, independent of age and sex.

The extent to which locus coeruleus structure related to memory performance in all participants are presented in Table 7 and Fig. 4. The whole locus coeruleus correlation analyses revealed that better (lower) recall variability was significantly related to higher restricted diffusion. The subdivision and hemisphere correlation analyses further revealed that this relationship was significant for restricted diffusion in the caudal subdivision and the right hemisphere of the locus coeruleus.

**Table 7:** Locus coeruleus structure relates to memory performance.

	Mean					Variability				
	Whole	Rostral	Caudal	Left	Right	Whole	Rostral	Caudal	Left	Right
MTC metrics										
Average	*0.21*	*0.20*	0.13	0.16	0.17	− 0.12	− 0.14	− 0.13	− 0.14	− 0.10
Maximum	0.08	n/a	n/a	n/a	n/a	0.18	n/a	n/a	n/a	n/a
DTI metrics										
MD	0.10	*0.19*	0.05	0.04	0.07	0.03	0.05	0.03	− 0.03	0.07
AD	*0.19*	*0.21*	0.10	0.07	0.16	− 0.02	− 0.02	< 0.01	− 0.04	0.05
RD	< 0.01	0.11	− 0.07	> − 0.01	0.04	0.06	0.09	− 0.01	> − 0.01	0.09
NODDI metrics										
Restricted	< 0.01	0.04	0.07	0.14	0.06	− **0.25**	− 0.17	− **0.22**	− 0.12	− **0.25**
Free	0.08	0.17	− 0.93	0.08	0.06	− 0.04	0.03	− 0.11	− 0.06	0.03

Pearson *R* statistics are provided for each partial correlation between memory performance (mean recall, recall variability) and each metric (MTC, DTI, NODDI) from the whole locus coeruleus (whole), its subdivisions (rostral, caudal), and hemispheres (left, right), controlling for age and sex. Significant Bonferroni corrected effects (*p* < 0.025) are bolded and non-significant trends (*p* < 0.05) are italicized.

### Relationships among locus coeruleus structure metrics

The extent to which locus coeruleus structure metrics were related are presented in Table 8. Results revealed significant positive relationships between metrics within each imaging modality (e.g., between average and maximum MTC). Higher free NODDI diffusion was also significantly related to higher diffusivity for all DTI metrics (MD, AD, RD).

**Table 8:** Correlation matrix for locus coeruleus structure metrics.

	Average	Maximum	MD	AD	RD	Restricted
MTC metrics						
Average						
Maximum	**0.39**					
DTI metrics						
MD	− 0.03	0.01				
AD	0.01	− 0.05	**0.74**			
RD	− 0.05	0.05	**0.87**	**0.31**		
NODDI metrics						
Restricted	0.15	0.10	− *0.20*	− 0.12	− *0.20*	
Free	< 0.01	0.12	**0.76**	**0.43**	**0.76**	**0.27**

Pearson *R* statistics are provided for each partial correlation between each pair of metrics from the whole locus coeruleus, controlling for age and sex. Significant Bonferroni corrected effects (*p* < 0.008) are bolded and non-significant trends (*p* < 0.05) are italicized.

## Discussion

The current study examined effects of age on locus coeruleus structure using a combination of magnetization transfer- and diffusion-weighted MRI in younger and older adults. Our approach extended prior work by examining age effects between younger and older adults as well as within older adults; including both MTC and DTI metrics and, for the first time, reporting NODDI metrics of locus coeruleus structure; comparing average and maximum MTC metrics; accounting for sex in all analyses; and reporting analyses by locus coeruleus subdivision and hemisphere. We were further interested in whether individual differences in locus coeruleus structure related to variability in memory performance. Key findings include that (1) maximum MTC in the locus coeruleus was more sensitive to differences between younger and older adults than average MTC, (2) DTI and NODDI metrics also showed significant, albeit smaller, age group differences, (3) males showed larger age group differences in MTC and NODDI metrics than females, (4) age group differences for DTI and NODDI, but not MTC, metrics varied across rostral and caudal subdivisions of the locus coeruleus, (5) age group differences were larger for MTC in the right than left locus coeruleus hemisphere, (6) only NODDI metrics were sensitive to effects of age within older adults, and (7) independent of age and sex, locus coeruleus structure (NODDI) related to variability in, but not mean, recall performance. These novel contributions to our understanding of the aging of locus coeruleus structure are detailed below.

When comparing age groups, significantly higher MTC was seen in older than younger adults for both the maximum and average metrics, comparable to at least some prior studies^25,33^. This finding is also consistent with there being a loss or reduction in locus coeruleus neurons with age^3–6^ c.f.^7^. Other studies that found no age group differences in locus coeruleus signal intensity have used a combination of maximum^34,35^ and average^36,37^ MTC, suggesting that metric type alone does not account for their discrepancies. We further showed, for the first time, that the age group difference was largest for the maximum relative to the average MTC metric, suggesting that future studies may improve their sensitivity to age group differences by using maximum MTC.

Relative to MTC, substantially fewer studies have examined aging of locus coeruleus structure using diffusion-weighted MRI, all of which modeled diffusion in each voxel as a single tensor. When using DTI, we observed significantly higher diffusivity in the locus coeruleus in older than younger adults and this age group difference was largest for AD relative to MD, but not significant for RD. Our age effects replicate at least one study that only looked at MD and similarly showed higher diffusivity in older than younger adults^33^, although it is inconsistent with prior work from our group that showed higher diffusivity in younger than older adults across all DTI diffusivity metrics^41,42^ and another group that only looked at MD and RD^37^. One explanation for this discrepancy is that our prior studies did not take sex into account, as was done here. Although Porat et al.^37^ considered sex and nonetheless found higher diffusivity in younger than older adults, albeit in some locus coeruleus hemispheres but not others across two datasets. Another, potentially more consequential, explanation is that the current study used a larger and less isotropic voxel (1.15 × 1.15 × 2.5 mm^3^) relative to prior work (0.95 × 0.95 × 1.0 mm^3^^41,42^; 1.7 mm^3^^37^) to increase signal-to-noise for NODDI fitting. Whereas the non-isotropic voxel should not affect the diffusivity measures as they are directionally invariant, we may be more susceptible to partial volume effects with adjacent white matter (superior cerebellar peduncle) and the fourth ventricle.

Prior diffusion-weighted MRI studies that examined effects of age on locus coeruleus structure had not modeled diffusion using multi-compartment approaches that may better capture microstructural properties in gray matter as they estimate compartments of diffusion that are tissue invariant. When using NODDI, we observed a non-significant trend for higher free diffusion in the locus coeruleus in older than younger adults, whereas the age group difference did not approach significance for restricted diffusion. Interestingly, whereas maximum MTC in the locus coeruleus was the single best predictor of age group, classification accuracy was slightly improved when adding DTI metrics to the model, especially AD, but not when adding NODDI metrics. This suggests that NODDI may be less sensitive to aging of the locus coeruleus than DTI diffusivity, which contradicts what has been observed in other gray matter structures, such as the hippocampus^44^. Another possibility is that we may have too low signal-to-noise ratios for such complex modelling in this deep brain structure, as some participants had to be excluded from the NODDI analysis because of issues with model fitting (i.e., free diffusion values of zero). Nonetheless, finding that DTI and NODDI metrics show significant, albeit smaller, age group differences, and that DTI diffusivity improves the ability of maximum MTC to predict age group, indicates that diffusion metrics should be considered in future studies of locus coeruleus aging.

Of the previous aging studies that also examined sex differences in locus coeruleus structure, all were focused on MTC, and only one found lower maximum MTC in females than males that was independent of age group^25^. Most other studies either did not test for sex differences^30,35^ or reported no significant sex effects^2,24,29,31^. In contrast, we observed significant effects of sex for all imaging modalities, with males having higher average MTC, lower DTI diffusivity, and higher NODDI diffusion relative to females. We further found interactions between age group and sex for all imaging modalities. That is, average MTC and AD were significantly, and MD showed a non-significant trend to be, higher in older than younger males, but not females; RD was significantly higher in younger than older females, but not males; restricted diffusion was significantly higher in younger than older males, but not females; and free diffusion showed no significant age group difference in either sex group. Given that these interactions between age and sex have been underreported, we note the importance for future studies to consider sex as a biological variable when examining effects of aging on locus coeruleus structure.

Whereas prior work using MTC in the locus coeruleus indicated that age effects may be more prominent in the rostral than caudal subdivision^24,35,38,39^, we did not find that the difference in average MTC between younger and older adults varied between the locus coeruleus subdivisions. This discrepancy may be due to differences in the way locus coeruleus is subdivided across studies, as it has been suggested that the rostral/caudal subdivisions may be overly simplistic and not reliably captured with current in vivo imaging approaches^15,54^. However, prior work finding significant effects have used both two^24,35,38^ and three^39^ subdivisions, indicating that the number of subdivisions alone cannot explain these findings. In line with this prior work, and with the notion that the rostral subdivision is more vulnerable to cell loss and reductions in cell density in aging, comparable to what is seen in Alzheimer’s Disease^28^, we did find interactions between age group and subdivision for the DTI metrics, with higher AD in older than younger adults that was larger in the rostral than caudal subdivision; higher MD in older than younger adults that was significant in the caudal, but not rostral, subdivision; and higher RD in younger than older adults that was significant in the rostral, but not caudal, subdivision. Although these finding contradicts our prior work showing comparable age group differences in locus coeruleus diffusivity in both subdivisions^42^, we previously used a much smaller sample (35 younger, 28 older) and did not account for sex. The current study further found non-significant trends for higher NODDI diffusion in older than younger females in the caudal subdivision, but in younger than older females in the rostral subdivision, with no difference across subdivisions in males. Thus, the different metrics and imaging modalities may be differentially sensitive to aging of locus structure across its subdivisions and interactions among these variables, as well as sex, warrants further investigation.

Most prior work using MTC in the locus coeruleus found no evidence that age effects differed in the left and right hemisphere^39,40^. Such findings are in line with postmortem literature that showed symmetrical aging of the locus coeruleus^5^. However, we found that the age group difference in average MTC was significantly larger in the right than left hemisphere. At least one prior DTI aging study also found larger age group differences in the right than left locus coeruleus^37^, although our age effects for DTI and NODDI metrics were comparable across the hemispheres. Future studies are needed to validate these findings by testing for hemispheric differences.

In contrast to the aforementioned results showing significant differences between younger and older adults for all imaging modalities, we did not find any significant relationships between chronological age and any measure of whole locus coeruleus structure within older adults. We did find one significant interaction between chronological age and sex for free diffusion in the left hemisphere, with older age relating to higher free diffusion in males and lower free diffusion in females. There were non-significant trends for older age relating to lower MTC (*p*s < 0.13), consistent with at least some prior studies^31,32^, and in the same direction as others that observed a decrease in locus coeruleus MTC with age after ~ 60 years old^2,24,29,30^. Looking across our extreme age group and within older adult findings for MTC, they are generally consistent with prior work reporting an inverted U-shaped function for locus coeruleus structure across the adult lifespan. However, age effects were significant and larger between younger and older adults for maximum than average MTC, but comparable across metrics within older adults, suggesting that future studies may improve their sensitivity to age effects by selecting the appropriate metric given their sample.

Independent of age and sex, we found that higher NODDI restricted diffusion in the locus coeruleus was significantly related to better (lower) recall variability, but not mean recall. Non-significant trends were also seen between higher average MTC and better (higher) mean recall, consistent with prior studies in older adults^2,12,35,36,45,46^; and between higher MD and AD and better mean recall, consistent with our previous findings within older adults^41,42^. Here, we extend prior work by demonstrating the sensitivity of NODDI metrics in the locus coeruleus to memory performance, and the sensitivity of variability in memory performance to locus coeruleus structure. Our significant finding is also consistent with the notion that locus coeruleus structure contributes to its function via noradrenergic signaling. Individual and age-related differences in locus coeruleus structure may influence moment-by-moment attention states that, in turn, affect consistency in (variability) task performance, as previously shown for a working memory task^47^. Recent animal studies have also demonstrated that a specific loss of locus coeruleus noradrenergic neurons was associated with worse memory performance^55^. Of note, our findings suggest that variability between trials may be more sensitive to degradation of locus coeruleus structure than average performance metrics (e.g., mean across trials) and should be considered in future work.

Interpretations of the current study are somewhat limited by the extreme age group and cross-sectional designs. Future research including middle-aged adults and repeated MRI sessions over time will provide a more detailed picture of locus coeruleus aging. Additionally, estimating diffusion in the locus coeruleus remains a challenge. As previously mentioned, we elected to use larger and less isotropic voxels (1.15 × 1.15 × 2.5 mm^3^) relative to prior work to increase signal-to-noise ratios for NODDI fitting, yet some participants were excluded for issues with model fitting (i.e., free diffusion values of zero). We also suspect that these larger voxels may be more susceptible to partial volume effects with adjacent white matter (superior cerebellar peduncle) and the fourth ventricle. Nonetheless, our findings demonstrate that diffusion-weighted MRI is sensitive to the effects of age on locus coeruleus structure and provides complementary information to the more commonly used magnetization transfer-weighted MTC metrics.

The current study aimed to bridge literatures that have examined age effects on locus coeruleus structure using different MRI modalities (magnetization transfer-weighted, diffusion-weighted) in different samples (between younger and older adults, within older adults) to identify which metrics are most sensitive to age and memory performance. When examining age effects between younger and older adults, we show that maximum MTC is the best predictor of age group, outperforming average MTC and both DTI and NODDI metrics that showed significant, but smaller age effects. We further show that age group differences in locus coeruleus structure vary with sex, subdivision, and hemisphere, and we encourage future studies to consider their contributions. Within older adults, however, there were no significant effects of age on any measure of whole locus coeruleus structure, just an interaction between chronological age and sex for free diffusion in the left hemisphere. Finally, we demonstrated that individual differences in locus coeruleus structure (NODDI restricted diffusion) significantly relates to memory performance, and that variability in performance may be as, if not more, sensitive than mean performance.

### Supplementary Information

Supplementary Information.

## Data Availability

The data in the current study are available from the corresponding author upon reasonable request.
