# Supplementary material for: Locus coeruleus contrast and diffusivity metrics differentially relate to age and memory performance
Source: Sci Rep. 2024 Jul 4;14:15372. doi: 10.1038/s41598-024-66238-z (PMC11224383; doi:10.1038/s41598-024-66238-z)
Supplement: Supplementary file 1 — Supplementary Information. [file 41598_2024_66238_MOESM1_ESM.docx]

**Locus Coeruleus Volume**

***Volume Metrics***

Volume of the locus coeruleus was measured as the number of voxels within the MT-GRE-aligned locus coeruleus region of interest with MTC values within four standard deviations from the mean of the pontine reference region (Langley et al., 2016, *Magnetic Resonance Materials in Physics, Biology and Medicine*). Individual differences in brain size were then corrected using the residual normalization method (Jack et al., 1989, *Radiology*). Intracranial volume was measured for each participant using the Estimated Total Intracranial Volume (eTIV_indiv_) generated by FreeSurfer (v.6.0; http://surfer.nmr.mgh.harvard.edu) and then averaged within younger participants (eTIV_mean_). The effect of brain size on locus coeruleus volume was estimated using the slope of the regression line between eTIV_indiv_ and Volume_raw_ within younger participants (*β*) to get estimates of these measures in the absence of age-related atrophy. Normalized volumes (Volume_norm_) were then calculated separately for each participant using the equation: Volume_norm_ = Volume_raw_ − β (eTIV_indiv_ − eTIV_mean_). The same procedure was used to estimate normalized volumes of locus coeruleus in the left and right hemispheres.

Total gray matter and white matter volumes were calculated for each participant using FSL’s FAST segmentation and normalized using the residual normalization method described above.

***Locus Coeruleus Volume Does Not Differ With Age or Sex***

An Age Group (younger, older) × Sex (male, female) one-way ANOVA on normalized volume of the whole locus coeruleus revealed no significant effects of Age Group or Sex, *p*s > 0.15. An Age Group × Sex × Hemisphere (left, right) mixed factorial ANOVA similarly revealed no significant effects of Age or Sex, *p*s > 0.30. Although, there was a significant main effect of Hemisphere, *F*(1, 126) = 96.6, *p* < 0.001, with larger volume in the left (7.5 ± 0.4) than right (4.7 ± 0.3) locus coeruleus. Within older adults, a regression analysis using Age, Sex, and their interaction as predictors yielded no significant effects, *p*s > 0.33. These findings are comparable to prior human MRI studies, which found no significant effect of age on locus coeruleus volume within older adults (Giorgi et al., 2021, *Brain Imaging and Behavior*) or between younger and older adults (Hammerer et al., 2018, *PNAS*).

Correlations between age and locus coeruleus in each hemisphere, controlling for sex, are shown below in Supplementary Figure 1.

***Locus Coeruleus Volume Does Not Relate to Memory Performance***

Partial correlations controlling for age and sex in the full sample revealed no significant relationships between either measure of memory performance (mean, variability) and normalized locus coeruleus volume (whole, left, right), *p*s > 0.13.

***Total Tissue Volume Did Relate to Memory Performance***

Partial correlations controlling for age and sex in the full sample revealed that better mean recall, *r* = 0.26, *p* = 0.004, but not recall variability, *p* = 0.076, was significantly related to larger (higher) normalized white matter volume. There was no significant relationship between either memory metric and normalized gray matter volume, *p*s > 0.14.

**Education**

It is not surprising that we found younger adults to have lower education than older adults as many of them are still in the process of completing their degrees. Because this measure does not accurately capture educational attainment in younger adults in the same way it does for older adults, we did not consider controlling for education in analyses with the younger age group.

***Controlling for Education Did Not Affect Non-Significant Effects of Age on Locus Coeruleus Structure Within Older Adults***

Effects of age and sex on locus coeruleus structure were re-assessed within older adults after controlling for years of education. Multiple regression analyses were conducted separately for each metric from each imaging modality using values from the whole locus coeruleus. For each model, chronological Age, Sex, Age × Sex, and Education were predictor variables and Metric was the observed variable.

Results revealed no significant effects, similar to what was reported in the manuscript when not controlling for education (Supplementary Table 1).

**Supplementary Table 1. Relationships to Age Within Older Adults**

|  | MTC | | | DTI | | | NODDI | |
| --- | --- | --- | --- | --- | --- | --- | --- | --- |
|  | Average | Maximum | MD | | AD | RD | Restricted | Free |
| *Whole locus coeruleus* | | | | | | | | |
| Age | -0.20 | -0.20 | 0.01 | | <0.01 | 0.01 | -0.18 | -0.04 |
| Sex | -1.35 | 0.10 | -0.58 | | -1.10 | <0.01 | -0.55 | -0.40 |
| Age × Sex | 1.34 | -0.08 | 0.46 | | 0.83 | 0.03 | 0.93 | 0.55 |
| Education | 0.12 | 0.16 | 0.15 | | 0.14 | 0.10 | 0.10 | 0.18 |

*Notes.* Standardized *Beta* coefficients are provided for each regression run on the whole locus coeruleus for each dependent metric (MTC, DTI, NODDI).

***Controlling for Education Did Not Affect Relationships Between Locus Coeruleus Structure and Memory Performance Within Older Adults***

Relationships between locus coeruleus structure and memory performance were re-assessed within older adults using separate partial correlations between each memory metric (mean recall, recall variability) and each metric from each imaging modality in the whole locus coeruleus, controlling for age, sex, and education. Significant effects survived Bonferroni correction for two comparisons per imaging metric (*p* < 0.025).

Results revealed only a non-significant trend for better (lower) recall variability relating to higher restricted diffusion, *r* = -0.24, *p* = 0.054, comparable to what was observed within older adults when only controlling for age and sex, *r* = -0.26, *p* = 0.036.

**Race**

***Controlling for Race Minimally Affected Age Group Differences in Locus Coeruleus Structure***

Effects of age and sex on locus coeruleus structure were re-assessed when controlling for race in all participants using separate Age Group (younger, older) × Sex (male, female) × Metric (MTC: average, maximum; DTI: MD, AD, RD; NODDI: restricted, free) mixed factorial ANCOVAs for each imaging modality (MTC, DTI, NODDI) with values from the whole locus coeruleus.

For the MTC and DTI analyses, the pattern of results remained the same as those reported in the manuscript when not controlling for race. For NODDI, however, the Age Group × Metric and Sex × Age Group × Metric effects were not significant when controlling for race. Thus, race may contribute to age group differences in locus coeruleus diffusion as measured with NODDI.

**Supplementary Table 2. Locus Coeruleus ANOVA Results Controlling for Race**

|  | MTC | DTI | NODDI |
| --- | --- | --- | --- |
| Age Group | **24.1** | **7.5** | 0.4 |
| Metric | **743.3** | **5209.5** | **11604.1** |
| Age Group × Metric | **5.6** | **12.6** | 1.8 * |
| Sex | 11.1 | **6.6** | **20.4** |
| Sex × Age Group | 1.6 | 0.7 | 0.3 |
| Sex × Metric | 2.0 | **3.2** | **14.3** |
| Sex × Age Group × Metric | 0.9 | 1.6 | 2.5 * |

*Notes. F* statistics are provided for each ANOOVA run on the whole locus coeruleus for each dependent metric (MTC, DTI, NODDI), controlling for race. Significant effects at *p* < 0.05 are bolded. * = effect differed from what was observed when not controlling for race.

***Controlling for Race Did Not Affect Relationships Between Locus Coeruleus Structure and Memory Performance***

Relationships between locus coeruleus structure and memory performance were re-assessed in the full sample using separate partial correlations between each memory metric (mean recall, recall variability) and each metric from each imaging modality in the whole locus coeruleus, controlling for age, sex, and race. Significant effects survived Bonferroni correction for two comparisons per imaging metric (*p* < 0.025).

Results revealed that better (lower) recall variability significantly related to higher restricted diffusion, *r* = -0.25, *p* = 0.009. Results also revealed a non-significant trend for better (higher) mean recall relating to higher average MTC, *r* = 0.20, *p* = 0.034, and higher AD, *r* = 0.19, *p* = 0.049. These findings are the same as those reported in the manuscript when not controlling for race.
